# Barriers and facilitators to implementing cancer prevention clinical decision support in primary care: a qualitative study

**DOI:** 10.1186/s12913-019-4326-4

**Published:** 2019-07-31

**Authors:** Melissa L. Harry, Anjali R. Truitt, Daniel M. Saman, Hillary A. Henzler-Buckingham, Clayton I. Allen, Kayla M. Walton, Heidi L. Ekstrom, Patrick J. O’Connor, JoAnn M. Sperl-Hillen, Joseph A. Bianco, Thomas E. Elliott

**Affiliations:** 1 Essentia Institute of Rural Health, 502 East Second Street, Duluth, MN 55805 USA; 2 HealthPartners Institute, 3311 E. Old Shakopee Road, Bloomington, MN 55425 USA; 3 Essentia Health – Ely Clinic, 300 W Conan Street, Ely, MN 55731 USA

**Keywords:** Cancer screening, Clinical decision support, Key informants, Pre-implementation, Primary and secondary prevention, Primary care, Qualitative

## Abstract

**Background:**

In the United States, primary care providers (PCPs) routinely balance acute, chronic, and preventive patient care delivery, including cancer prevention and screening, in time-limited visits. Clinical decision support (CDS) may help PCPs prioritize cancer prevention and screening with other patient needs. In a three-arm, pragmatic, clinic-randomized control trial, we are studying cancer prevention CDS in a large, upper Midwestern healthcare system. The web-based, electronic health record (EHR)-linked CDS integrates evidence-based primary and secondary cancer prevention and screening recommendations into an existing cardiovascular risk management CDS system. Our objective with this study was to identify adoption barriers and facilitators before implementation in primary care.

**Methods:**

We conducted semi-structured interviews guided by the Consolidated Framework for Implementation Research (CFIR) with 28 key informants employed by the healthcare organization in either leadership roles or the direct provision of clinical care. Transcribed interviews were analyzed using qualitative content analysis.

**Results:**

EHR, CDS workflow, CDS users (providers and patients), training, and organizational barriers and facilitators were identified related to Intervention Characteristics, Outer Setting, Inner Setting, and Characteristics of Individuals CFIR domains.

**Conclusion:**

Identifying and addressing key informant-identified barriers and facilitators before implementing cancer prevention CDS in primary care may support a successful implementation and sustained use. The CFIR is a useful framework for understanding pre-implementation barriers and facilitators. Based on our findings, the research team developed and instituted specialized training, pilot testing, implementation plans, and post-implementation efforts to maximize identified facilitators and address barriers.

**Trial registration:**

clinicaltrials.gov, NCT02986230, December 6, 2016.

**Electronic supplementary material:**

The online version of this article (10.1186/s12913-019-4326-4) contains supplementary material, which is available to authorized users.

## Background

Primary and secondary cancer screening and prevention is commonly addressed in primary care practice in the United States [1]. However, primary care providers (PCPs) may juggle complex patient needs and conditions with competing demands, all during time-limited visits. Introducing clinical decision support (CDS) into the electronic health record (EHR) provides an avenue for triggering cancer screening and prevention reminders for PCPs and patients, supporting the Chronic Care Model [2]. Computerized CDS systems can perform multiple tasks. These include identifying patients who could benefit from evidence-based interventions, presenting prioritized evidence-based prevention and treatment options to both patient and PCP at the point of care, and facilitating efficient ordering of recommended treatments, screening tests, medications, or referrals [3, 4]. CDS systems can also be programmed to include cancer risk calculators and decision aids. Decision aids provide written information on the type of cancer, screening or treatment options, risks, benefits, and questions or statements designed to help patients decide how to proceed.

Shared decision-making between patients and providers may benefit from encompassing three areas supported by decision aids [5]. These include presenting choices to patients, listing available options, and having providers confer agency to patients by providing this information and supporting patients in the decision-making process [5]. A Cochrane Review of 105 studies on different types of decision aids found that they increased patient knowledge on available options, associated risks, and personal preferences. Decision aids also reduced decisional conflict, were associated with satisfaction rates the same as or greater than non-decision aid use, as well as may improve patient and provider discussions [6]. However, use of decision aids slightly lengthened appointment time by a median of 2.6 min, and more research is needed on patient decision adherence and cost-effectiveness [6].

Although CDS systems have been available in some form for more than five decades, and designs adapt along with clinical practice, provider adoption is still an issue [7]. Literature on the effectiveness of CDS systems suggests barriers and facilitators to use and acceptance in clinical settings. In a meta-regression of 162 computerized CDS systems previously studied, improvements in either patient outcomes or care processes (e.g., provider activities) were seen in just 58% of trials [8]. CDS systems had higher odds of improving patient outcomes if: the systems targeted both patients and providers; were developed by the respective study authors (these studies may have been affected by positive publication bias); or required users to input reasons for not adhering to recommendations into the CDS system [8]. However, compared with separate CDS systems, CDS recommendations embedded in electronic systems like EHRs had significantly lower odds of improving either patient outcomes or care processes [8]. Alert fatigue, which is associated with both CDS and EHRs in the literature [7], was suggested as a likely contributor to this finding; CDS can become just another of the EHR alerts that already inundate providers and may ultimately be ignored [8].

### Integrating cancer prevention CDS and decision aids into a cardiovascular risk management CDS system

To assess the effectiveness of incorporating cancer screening and prevention CDS into primary care, we updated a cardiovascular risk management CDS system used at Essentia Health, an upper Midwestern healthcare system in the United States [9]. We did so as part of a National Cancer Institute-funded, pragmatic, three-arm, 36-clinic, cluster-randomized control trial (RCT) (with three clinics randomized together due to shared PCPs, for 34 randomization units). The cardiovascular CDS system was already part of two studies of patients with either prediabetes/diabetes or serious mental illness. We added new primary (human papillomavirus [HPV] vaccination) and secondary (breast, cervical, colorectal, lung) cancer prevention and screening CDS recommendations for all patients in intervention clinics eligible based on the United States Preventative Services Task Force (USPSTF) and the Centers for Disease Control and Prevention’s Advisory Committee on Immunization Practices guidelines [10–14]. The cardiovascular CDS also has tobacco cessation and obesity management components. The cancer prevention CDS focuses on these two areas as well, as both relate to primary cancer prevention. The CDS is a web-based application integrated into the healthcare system’s EHR and available only to study intervention clinics. The web-based application reads data from the EHR, runs algorithms, and returns personalized patient recommendations in print and electronic form.

The 24 cardiovascular CDS intervention clinics were randomly assigned to one of two intervention arms to receive either: the new CDS system with both cardiovascular risk reduction and cancer prevention aims; or both the new CDS system and decision aids for HPV vaccination (parent and adult versions) and breast, colorectal, and lung cancer screening. The decision aids will be reported on separately. The CDS in both arms has also been programmed to include widely used and publicly available breast, colorectal, and lung cancer risk calculators [15–18]. The recommended workflow is for clinic rooming staff to print the patient and provider versions of the CDS, place the provider version on the exam room door, and give the patient version, along with any decision aids in one intervention arm, to the patient. A third control arm receives cancer prevention and screening care as usual. Of note, clinic rooming staff are individuals, like clinical or medical assistants and nurses, who take the patient from the waiting room to the exam room. Rooming staff begin the visit by assessing patients’ height, weight, blood pressure, temperature, other vital signs, and current medications before the PCP enters the room.

## Methods

### Study purpose and guiding research question

The purpose of this study was to identify pre-implementation barriers and facilitators for the cancer prevention components of the integrated CDS system. We did so by interviewing healthcare system key informants (leadership and cardiovascular CDS intervention clinic PCPs and rooming staff) before implementation of the cancer prevention CDS. Our guiding research questions, some of which were adapted from the Consolidated Framework for Implementation Research (CFIR) [19], included understanding: (1) factors that facilitate or hinder key informant support for the intervention; (2) key informant knowledge and beliefs about the intervention and tension for change; (3) the relative advantage(s) of the intervention compared with other interventions currently available in the EHR; (4) relevant organizational culture norms and values related to cancer prevention and screening; (5) factors that may foster adoption from a key informant perspective; (6) related external policies and incentives; and (7) the implementation climate.

### Qualitative approach and research paradigm

In this study, we used qualitative content analysis in answering our guiding research questions [20]. Our data collection and analysis were also informed by the CFIR as a guiding framework. The CFIR encompasses five domains and numerous constructs adapted from 19 other implementation theories and frameworks [19]. We chose the CFIR for its flexibility in assessing implementation barriers and facilitators; researchers are encouraged to choose relevant constructs rather than applying the framework as a whole [19]. Others have used the CFIR in assessing barriers to and facilitators of adapting a mail-based colorectal cancer screening program to a study site before implementation [21]. A web page also provides qualitative CFIR interview questions that researchers can adapt [22]. Our work falls within the constructivist/interpretivist research paradigm, in which knowledge is considered subjective and reality socially constructed [23]. While we use the CFIR framework in this study as a guiding framework, we made every attempt to retain the subjective meaning expressed by key informants in our presentation of study results.

### Researcher characteristics and reflexivity

TE is the lead principal investigator for the main cancer prevention CDS RCT with HealthPartners Institute. DS is the Essentia Health site principal investigator for the prediabetes cardiovascular and cancer prevention CDS RCTs. Both TE and DS have working relationships with some of the key informants who took part in this study. JB is a site co-investigator and a primary care physician in a CDS intervention clinic. He was also co-director of primary care at Essentia Health when the interviews were conducted and suggested healthcare system leaders for key informant interviews. PO and JS-H are co-investigators with HealthPartners Institute for the cardiovascular and cancer prevention CDS RCTs and co-led the design team in developing and implementing the CDS. HE is a project manager on the cancer prevention CDS study with HealthPartners Institute and has helped design and develop the CDS. Neither TE, DS, JB, PO, JS-S, nor HE took part in the key informant interviews or qualitative data analysis for this paper. MH was the site project manager for the cancer prevention CDS RCT for Essentia Health when the interviews were conducted and is currently a site co-investigator. HH-B is a site research project coordinator for the cardiovascular and cancer prevention CDS studies at Essentia Health. MH and HH-B had no previous relationships with study participants. CA is the site project manager for the prediabetes cardiovascular CDS RCT and member of the cancer prevention CDS RCT team at Essentia Health. While CA has ongoing work relationships with clinic managers and Essentia Health primary care leaders, he was not part of interviews conducted with these individuals. KW is the site project manager for the cancer prevention CDS RCT at Essentia Health; she was not a study team member when the interviews were conducted, but has ongoing relationships with clinic managers and healthcare system primary care leaders in her role as project manager. AT is a project manager on the cancer prevention CDS RCT with HealthPartners Institute and was not a study team member when the interviews were conducted. MH, TE, DS, HH-B, HE, PO, JS-H, JB and CA developed the interview guides. MH, HH-B, and CA conducted key informant interviews, and MH, KW, and AT analyzed qualitative data for this paper.

### Study setting/context

The cancer prevention and cardiovascular CDS is in use at 24 primary care intervention clinics at Essentia Health, a large and predominately rural integrated healthcare system with more than 70 clinics and 15 hospitals in four upper Midwestern states (Idaho, Minnesota, North Dakota, and Wisconsin). Clinics in this study are in Minnesota, North Dakota, and Wisconsin. Idaho clinics were not included due to their lack of an EHR. The cancer prevention CDS was designed to help improve rates of HPV vaccination and breast, colorectal, lung, and cervical cancer screening and prevention in the healthcare system.

### Sampling strategy

Healthcare system leadership key informants were initially identified by the healthcare system’s co-director of primary care, also a co-principal investigator on this study (JB). We chose to interview leaders because they have input into and oversight over clinic operations. This includes approval for adopting new interventions systemwide, such as the cancer prevention and cardiovascular CDS system. Leadership can also be champions for interventions. Using snowball sampling, leadership key informants recommended additional leadership interviewees.

PCPs with past experiences with the cardiovascular CDS and current practice in an intervention clinic were invited to take part in an interview using purposive sampling. In addition, we recruited PCPs through intervention clinic managers and other key informants and by schedule availability. Intervention clinic managers recommended clinic rooming staff for interviews.

### Ethical issues pertaining to human subjects

A waiver of documentation of informed consent was granted by the Essentia Health Institutional Review Board. This waiver was granted because the care recommendations in the cancer prevention CDS intervention encompass evidence-based care already recommended in current US national and regional clinical guidelines. Interviews with key informants involved no more than minimal risk to interviewees. All interviewees verbally consented to taking part in the interviews.

### Data collection methods

Semi-structured interviews were conducted in person or using videoconferencing from June to September 2017, when data saturation was reached. Interviews were conducted as a dyad by the site project manager at that time (MH) and site research project coordinator (HH-B). An additional site team member (CA) also conducted interviews as backup for the research project coordinator. All interviews started with a roughly five-minute PowerPoint presentation by MH, which included sharing mock-up printouts of the CDS patient and provider interfaces and sample decision aids. Then the dyad received verbal consent from the interviewee for conducting the interview. Interviews were completed six to 11 months before the cancer prevention CDS intervention was implemented on May 1, 2018. Interviews lasted an average of 35 min (range: 20 to 60 min). Recorded interviews were professionally transcribed and error checked for accuracy. Any interviewer notes were added to the top of the transcribed interview documents before data analysis. Interviews were deidentified and stored in a secure healthcare system folder with access limited to Essentia Health study personnel.

### Data collection instruments

We developed similar but separate semi-structured interview guides for leaders and PCPs/rooming staff (Additional file 1). These guides were informed by the CFIR [19] and the implementation of the prediabetes/diabetes cardiovascular CDS RCT study. Questions were on topics such as cancer prevention and screening, the cardiovascular CDS, the cancer prevention components of the integrated CDS system, shared decision-making, and decision aids. Furthermore, all key informants were asked questions from the following CFIR domains: Characteristics of Individuals (Knowledge and Beliefs about the Intervention), Inner Setting (Implementation Climate; Tension for Change), and Intervention Characteristics (Relative Advantage) [19]. Leadership key informants were also asked about the organization’s cancer prevention priorities and the CFIR Outer Setting domain (External Policies and Incentives) [19]. While clinic PCPs and rooming staff were not asked about Outer Setting or organizational priorities, they did receive additional questions on the functionality of the new cancer prevention components of the cardiovascular CDS system in practice, their perceptions of shared decision-making (PCPs only), and how they used the cardiovascular version of the CDS system. Sample probing questions were included in the guides, but interviewers could also ask impromptu probes based on key informant responses. We pilot tested the interview guides with a healthcare system leader and a physician not included in the sample reported here.

### Data analysis

Data were analyzed in four phases using open coding steps adapted from grounded theory and qualitative content analysis [20]. First, MH coded the interviews using open coding. She then developed a coding frame focusing on barriers and facilitators that emerged from open coding, and recoded the interviews using this coding frame. Next, AT was brought onto the study as a second coder. AT coded all interviews separately with a coding frame of overarching barriers and facilitators. MH and AT then compared coding in small batches and came to agreement when barrier or facilitator coding differed. As an additional coding reliability check, KW coded a subset of interviews (two leaders, two PCPs, and two rooming staff) [20] using the codes used by MH and AT. KW coded the interviews in an iterative fashion, working with MH to come to consensus on the barriers and facilitators, which included collapsing some similar codes to enhance reader comprehension (e.g., combining separate codes for the CDS being seen as an improvement over current EHR alerts and tools). Finally, MH and KW used the CFIR as a coding frame for coding the final list of barriers and facilitators. While we included codes for CFIR domains and constructs used in the interview guides, we also coded key informant responses to other CFIR constructs, if applicable. Continuing to employ a consensus approach, MH and KW agreed on all CFIR coding. Techniques to enhance the trustworthiness of the data included using multiple coders, sharing a summary of findings with key informant interviewees for member checking (none responded with comments), and keeping an audit trail of steps taken in all analyses.

## Results

Interviews were conducted with 28 key informants employed by the healthcare system. The sample included 13 healthcare system leaders, 13 PCPs (six physicians, two nurse practitioners, one physician assistant, and four registered nurses [RNs]) and two rooming staff (one clinical assistant and one licensed practical nurse) working in healthcare system clinics currently using the cardiovascular CDS. Women made up 41% of the sample.

Informants described barriers to and facilitators of CDS implementation and adoption related to constructs in the Intervention Characteristics, Outer Setting, Inner Setting, and Characteristics of Individuals CFIR domains. Table 1 presents the primary codes and count data differentiated by CFIR domain and construct, as well as whether codes represented barriers to or facilitators of cancer prevention CDS implementation and adoption. Only relevant CFIR domains and constructs were coded and presented here.

**Table 1:** Key informant-identified barriers and facilitators to the implementation and adoption of the cancer prevention CDS by CFIR domain and construct (*n* = 28)

CFIR Domains & Constructs	Barriers	Count	Facilitators	Count
I. Intervention Characteristics				
B. Evidence Strength and Quality	Concern about inaccuracy or conflicting CDS recommendations compared to healthcare system recommendations^a^	9	CDS follows USPSTF recommendations	3
C. Relative Advantage			CDS improvement over current EHR alerts and tools	18
			Potential for time savings	7
			CDS similar to current EHR alerts and tools	5
D. Adaptability			Optimize CDS integration into clinic workflow	22
G. Design Quality and Packaging	CDS duplicates or complicates care	11		
II. Outer Setting				
A. Patient Needs and Resources	Financial costs to patients	11	Patient self-education^a^	10
	Patient socioeconomic disparities	5	Patients controlling own health^a^	9
	Patient transportation issues	4	Organization increasing PCP patient visits from 18 to 22 a day^a^	8
			Reminders to patients^a^	5
			Repeated exposure for patients^a^	5
			Focus on prevention over crisis or acute^a^	5
			Lung cancer screening^a^	3
D. External Policy and Incentives			Positive impact on quality metrics^a^	11
III. Inner Setting				
C. Culture			Alignment with institutional aims^a^	16
D. Implementation Climate				
1. Tension for change	PCP time limitations^a^	25	PCP time limitations are manageable	5
	Alert fatigue (PCPs and/or patients)	25		
2. Compatibility	Not appropriate for acute visits – annual only	9	CDS appropriate for many visit types	5
	Not everything in the EHR is accurate or easy to find	9	Others than PCPs using CDS:	22
	RN CDS use issues (e.g., not appropriate for all RN visit types, RN roles can vary by clinic, RN shortage in healthcare system)^a^	5	RNs using CDS in general	22
			RNs using CDS during Medicare annual Wellness visits	14
			RNs using CDS alongside other PCPs	4
			Clinic rooming staff using CDS	4
			Get CDS printouts to patients before provider (e.g., pre-visit use, before PCP enters room)	18
			Alignment with institutional aims^a^	16
			Institution-wide streamlining of EHR alerts	9
			Team model of care	8
3. Relative Priority	Seen as just another initiative	10		
	Organization increasing PCP patient visits from 18 to 22 a day^a^	8		
	Lack of institutional initiative prioritization	3		
4. Organizational Incentives and Rewards			Positive impact on quality metrics^a^	11
E. Readiness for Implementation				
2. Available Resources	PCP time limitations^a^	25		
	Not all clinics have color printers - looks better in color	6		
	RN CDS use issues (e.g., not appropriate for all RN visit types, RN roles can vary by clinic, RN shortage in healthcare system)^a^	5		
	PCP shortage/burnout	4		
	Too few printers	3		
	Clinic rooming staff – already crunched for time	2		
3. Access to Knowledge and Information	Does not recall receiving cardiovascular CDS training	8	Providing in-person training on the CDS	16
	E-learning not always effective	6	E-learning or webinars are acceptable	6
			Provide PCPs with supporting CDS evidence	6
			Focus on workflow in training	5
			Provide multiple learning points	4
IV. Characteristics of Individuals				
A. Knowledge and Beliefs about the Intervention	Concern about inaccuracy or conflicting CDS recommendations compared to healthcare system recommendations^a^	9	Patient self-education^a^	10
	PCP distrust of HPV vaccine or cancer risk calculators	3	Patients controlling own health^a^	9
			Reminders to patients^a^	5
			Repeated exposure for patients^a^	5
			Focus on prevention over crisis or acute^a^	5
			Lung cancer screening^a^	3

CFIR - Damschroder et al. [19]. Sample size (n) refers to number of informants interviewed. Count refers to the number of informants that mentioned a specific barrier or facilitator

^a^Code fit with two CFIR constructs. Could also be a barrier for one construct and a facilitator for another

### Intervention characteristics

#### Evidence strength and quality

Nine key informants stressed that inaccurate CDS recommendations or recommendations that conflict with the healthcare system could be barriers to CDS use: (ID 530) “Because if it’s pulling inaccurate information or there’s three [EHR alerts] that are firing about the same thing and there’s so much overlap, that’s going to be a point of frustration and ultimately, people will just wash their hands of it.” (Also coded as a barrier under Characteristics of Individuals, Knowledge and Beliefs about the Intervention.) However, three informants mentioned that a benefit of the CDS is its use of the USPSTF recommendations for cancer screening.

#### Relative advantage

The cancer prevention CDS was seen to have multiple advantages over the current healthcare system’s EHR. Eighteen informants described the CDS as an improvement over current EHR alerts and tools: (ID 528) “I think it compares favorably, because what we get is a[n] [EHR] alert saying somebody’s overdue for their colonoscopy. And this would enable you to have a better, more formatted shared decision-making process around cancer screening.” Compared with other EHR tools, the CDS was seen as offering the ability to place recommended orders through the CDS interface, provide paper printouts directly to patients for their review, and bring health maintenance “due” alerts to the fore. Seven informants also thought the CDS could save time at clinic visits. Five informants described the CDS as very similar to tools and alerts available in the EHR, but found this as a facilitator to adoption rather than a barrier.

#### Adaptability

Twenty-two informants recommended optimizing the integration of the CDS into clinic workflow, which could vary by clinic. Making the CDS easy to use was important: (ID 532) “I think just the time issue, so ease of use…it’s just making the process as easy as possible.” Informants also said the research team needed to understand how the CDS would affect clinic workflow, particularly where in the workflow the CDS printouts could be presented to patients most effectively and efficiently with the least impact on PCPs. While adapting the CDS to work with clinic workflows was seen as a facilitator for CDS use, a lack of integration into existing workflows could be a barrier, potentially limiting CDS use.

#### Design quality and packaging

Eleven informants worried that the CDS could duplicate, and thereby possibly complicate, patient care: (ID 529) “Is it going to be duplicating everything that they’re [clinic staff who identify patients due for screenings before a visit] doing already, I wonder?” (ID 132) “It just might be too much information, and then it dilutes out the message.”

### Outer setting

#### Patient needs and resources

Eleven informants raised issues about patients having limited health insurance coverage, high deductibles, facing risks associated with screenings, and dealing with the potential costs of screening in the presence of abnormal findings: (ID 545) “‘But the Affordable Care Act says it’s supposed to be paid for.’ Well, yeah, the screen part is, but if they have to do a biopsy, and suddenly they have big bills to pay, and it’s for me to negotiate that as the primary care person.” Five informants also described issues related to patient socioeconomic disparities affecting cancer prevention and screening uptake more generally, as well as four noting patients can experience difficulty securing transportation to appointments or screening tests.

Considering patient needs, the healthcare system’s move to improve patient access by increasing the number of patients that PCPs see in a day from 18 to 22 was seen as a potential facilitator for CDS use by eight informants. (Also coded as a barrier for Inner Setting, Implementation Climate, Relative Priority.) Ten informants said that the CDS would be helpful for patient education, and nine said it would help patients control their own health: (ID 541) “Patients can’t retain all this stuff...this gives them something else to walk out with and think about.” Five also talked about the CDS being a good reminder for patients that also offers repeated exposure to cancer prevention and screening opportunities. The focus on prevention over acute or crisis care was a positive for five informants, with three informants identifying lung cancer screening as potentially very important for patients: (ID 535) “To me, lung’s the biggest one. That’s the opportunity that’s probably biggest.” (These patient-focused facilitators were also coded as facilitators under Characteristics of Individuals, Knowledge and Beliefs about the Intervention.)

#### External policy and incentives

Eleven informants said that the CDS could improve cancer prevention and screening clinical quality metrics at the healthcare system, which are tied to improving both patient health and PCP compensation: (ID 520) “Our providers are going to be looking for anything that will make their job easier to meet their [quality metric] initiatives because that’s how they get paid. I mean, let’s be honest.” (Also coded as a facilitator for Inner Setting, Implementation Climate, Organizational Incentives and Rewards.)

### Inner setting

#### Culture

Sixteen informants expressed that the CDS aligned well with the healthcare system’s aims:

(ID 518) “Our aspirational aim is to achieve health and vitality in our community and that’s about one patient at a time, making sure that their health and well-being is of most importance and we’re called to make a healthy difference. So, I’d say it’s [cancer prevention and screening] at the top of their priorities.”

(Also coded as a facilitator for Inner Setting, Implementation Climate, Compatibility.)

#### Implementation Climate: Tension for change

PCP time limitations were a major concern for 25 informants. (Also coded as a barrier for Inner Setting, Readiness for Implementation, Available Resources.) PCPs are being asked to do more with less time, including seeing more patients in a workday, making some PCPs wonder how to fit the CDS into the visit, as described by one PCP who was representative of others:

(ID 542) “Do we make it part of the appointment and give ourselves a little more time so we can discuss it? That’s the tough part, just trying to work it in, because you’re already going over on most of your appointment slots anyway.”

However, five of the same 25 informants who mentioned PCP time constraints also said that this limitation would not directly affect them, would only be a limitation until PCPs learned the CDS tools, or should and could be addressed: (ID 522) “If we commit to patient-centered care, then you take the time to have the discussion with the patient, the pros and the cons, and that takes time.”

Alert fatigue was an overarching theme described by 25 informants. Many informants reported that alerts are often ignored, clicked through, or simply not used. A few PCPs mentioned the potential for patient fatigue with the CDS if it were to trigger frequently. Some informants said CDS use could be negatively affected by alert fatigue, as described by one leadership PCP:

(ID 539) “Everybody gets tired. And then especially when they’re just at [a] hard stop, that gets old fast, because I think, I fear that we just end up doing whatever we can to get rid of it instead of really addressing it.”

#### Implementation Climate: Compatibility

Nine informants said that the CDS may be more appropriate for yearly visits like physicals and Medicare annual wellness visits [24] (Medicare is the government health insurance for US citizens or permanent residents aged 65 and older, some people younger than 65 with disabilities, and those with end-stage renal disease [25]), because the limited time providers have with patients is already crunched by prioritizing patient needs, alert fatigue, the organizational push to increase patient access by increasing the number of patients PCPs see in a day, and competing initiatives affecting primary care. As one typical leadership PCP stated:

(ID 526) “Yeah. But it isn’t going to pop up for an acute visit, is it? [Interviewer: “It will, in primary care, yes.”] ‘Oh, great’, said somewhat sarcastic[ly] [laughter]. So if I come in with, ‘Doctor, I think I broke my ankle this morning at nine o’clock when I fell off a ladder.’ ‘Well, we’ll look at that in a minute. First, let me talk to you about—’ ‘They’re not listening’ [laughter].”

However, five informants said that the CDS was appropriate for multiple visit types: (ID 523) “I think it should fire whenever they come, because we’ve got them.”

Nine informants mentioned issues specific to the EHR, such as difficulty finding both outside records from other healthcare institutions and in-house colonoscopy report results in the EHR. Specifically, outside records are often scanned in and could not be easily searched in the EHR at the time of our study, which may limit the utility of the cancer prevention CDS in locating these records. Also, in-house colonoscopy reports include results buried in the report rather than diagnoses easily available in the EHR. One PCP informant said it would be very beneficial if the cancer prevention CDS could search these reports for key phrases related to colonoscopy reports.

Most informants (*n* = 22) recommended having other team members, such as RNs or rooming staff, go through the CDS printouts with patients rather than just the PCPs alone. As one PCP with a leadership role said:

(ID 536) “I’m already 20 minutes behind. I don’t have five or 10 minutes to try and walk through this sort of stuff. That’s where the whole team concept comes in and somebody else that does have that time so they can actually give it the justice that it deserves to the importance of it. Because this is important stuff. This is really important stuff. And we don’t want to shortchange it. And it’s going to get shortchanged if we put this all on the clinician’s shoulders.”

Twenty-two informants endorsed RNs using the CDS, including 14 specifying RN use in prevention-focused Medicare annual wellness visits, in which the healthcare system is increasingly incorporating RNs. Echoing others, one leadership informant wondered why RNs could have the CDS in Medicare annual wellness visits, but not other visits, as RNs have the time and skill set to have those types of conversations with patients. As one PCP informant put it: (ID 523) “They went into their nursing school to do patient care. And that’s what this is, at the highest level, really.” Four informants recommended that RNs use the CDS along with other providers, as RNs cannot order low-dose chest computed tomographic scans for lung cancer screening, for example. Several barriers to RN use were also reported. (Also coded as barriers for Inner Setting, Readiness for Implementation, Available Resources.) Three informants, including one RN, did not think the CDS would be appropriate for all RN visit types, since some visits are short and focused (e.g., allergy shots). Still, informants did seem to think the CDS was appropriate for other RN ancillary visit types (e.g., hypertension follow-ups, diabetes care, other chronic disease management visits). However, the CDS only fires for office and Medicare annual wellness visits. It does not fire for the ancillary visits that many RNs perform. RN visit types and roles can also vary by clinic, according to three informants. Furthermore, two informants described a shortage of RNs in the healthcare system:

(ID 536) “RN[s] right now is where we’re lacking. And there’s so much we could do with an RN. When we talk about access, improving [patient] access [to primary care], and that sort of stuff, we are not utilizing RNs like we could to be able to improve access. We just don’t have-- and it’s not that we’re not utilizing them because we’re not giving them the opportunity. We’re not utilizing them because we don’t have enough RNs to do what we want them to do. We need more RNs.”

Regarding clinic rooming staff (e.g., certified medical assistants, clinical assistants, licensed practical nurses) using the CDS, three leaders and one PCP informant supported clinic rooming staff using the CDS with patients, including by (ID 528) “walk [ing] patients though [the CDS or] getting them started,” as well as helping patients prioritize what they would like to discuss with their PCP on the CDS handouts.

Eighteen informants talked generally about getting the CDS printouts to patients before seeing their PCPs, which is part of the recommended workflow: (ID 534) “People are way more sophisticated today as far as knowing about stuff, so it doesn’t bother me if they read a little bit beforehand. It might save us some time if they have some questions that way.”

In addition to the 16 informants that noted the cancer prevention CDS aligned with institutional aims (see Inner Setting, Culture), nine informants brought up how the healthcare system recently instituted changes in EHR alerts to streamline alert firing and reduce alert fatigue. A move towards using a team model of care in primary care was also considered a potential facilitator to CDS use by eight informants:

(ID 535) “We’re building new team models in primary care, so we’ve got a lot of work going into what’s the [nurse practitioner] role, what’s the nurse’s roles, what’s the [clinic assistant/rooming staff] role, what’s the provider role. So I think it’s still in a state of evolution, but as we try to work through that, different people will take on different roles with the patient to go through things like [the CDS].”

#### Implementation Climate: Relative Priority

Ten informants perceived the CDS as potentially being viewed as just another initiative, one of many already affecting overworked PCP schedules. Eight informants brought up the healthcare system’s push to increase patient access to primary care, specifically by increasing PCP visits from 18 to 22 a day (see Outer Setting, Patient Needs and Resources) as a potential barrier, as the CDS could further affect PCP time limitations:

(ID 533) “I think it’s really important you get [PCPs’] input, and they’re going to want to know, honestly, how much time is this going to take? We’ve asked them a lot. We’ve asked a lot out of our primary care docs. We’ve asked a lot out of them to increase access, to increase throughput, which is to say, to see more patients, to be more productive.”

Three informants also talked about a lack of prioritization when rolling out initiatives at the healthcare system level: (ID 531) “There’s so many different initiatives and so many different changes that it’s hard to keep them all straight if you’re doing four or five changes at once.”

#### Implementation Climate: Organizational Incentives and Rewards

The potential for positive impact on clinical quality metrics around cancer prevention and screening was seen as a facilitator of CDS use by 11 informants (see Outer Setting, External Policy and Incentives).

#### Readiness for Implementation: Available Resources

The scarcest resource described by 25 informants was PCP time (see Inner Setting, Implementation Climate, Tension for change). Four informants pointed out that a shortage of PCPs and high levels of provider burnout in general are important barriers for the team to consider. While many informants recommended that RNs use the CDS, barriers to wide RN use of the CDS in the healthcare system still exist (see Inner Setting, Implementation Climate, Compatibility). Furthermore, one leader informant practicing in a non-study clinic and another practicing in an intervention clinic pointed out that clinic rooming staff were already crunched for time; the CDS would just add more to their plate. Some informants also described printer issues related to printing the patient and provider versions of the CDS. These issues included too few printers in some clinics and a dearth of color printers in general (key informants liked how the mock-up CDS print-outs looked with color ink).

#### Readiness for Implementation: Access to Knowledge and Information

Of importance, eight cardiovascular CDS clinic informants reported that they did not receive or recall training on the cardiovascular CDS intervention the previous year. One PCP reported receiving paper training after implementation of the cardiovascular CDS and thought the paper training was effective, if not timely. Six informants did not like e-learning, the primary method of training used by the healthcare system, or found it to be ineffective. Sixteen informants recommended in-person training on the CDS at intervention clinics to increase uptake:

(ID 519) “I think your best way to approach it personally would be, in a family practice setting at least, is for you guys or whoever is bringing it out to come to both a section meeting [PCPs] and a staff meeting [nurses and rooming staff].”

Six informants did find e-learning or webinars acceptable methods for training. The same number emphasized that the research team should provide supporting evidence in the training on why PCPs should use the CDS. Five also thought training should focus on workflow, and four thought the research team should use multiple training modes or learning points.

### Characteristics of individuals

#### Knowledge and beliefs about the intervention

Of note, two PCPs expressed doubts about the efficacy of the HPV vaccine, and another expressed doubt about cancer risk calculators and similar tools. Nine informants were also concerned about the possibility of inaccurate or conflicting CDS recommendations (see Intervention Characteristics, Evidence Strength and Quality). Informants also saw the CDS as providing numerous benefits to patients (see Outer Setting, Patient Needs and Resources).

## Discussion

CDS systems may aid PCPs with cancer prevention and screening in busy primary care settings. In this study, we interviewed healthcare system leaders, PCPs, and clinic rooming staff key informants before implementation of a cancer prevention CDS in a preexisting cardiovascular risk management CDS system within a pragmatic, clinic cluster-RCT. Key informants identified numerous barriers to and facilitators of a successful implementation and high use of cancer screening and prevention CDS elements. Barriers and facilitators related to a number of CFIR Intervention Characteristics, Outer Setting, Inner Setting, and Characteristics of Individuals domains and constructs [19]. When implementing pragmatic interventions like the cancer prevention CDS, it is important for researchers in healthcare systems to identify and understand potential internal and external adoption barriers and facilitators. Conducting healthcare system employee key informant interviews pre-implementation, as well as applying an implementation framework like the CFIR, allowed our research team to identify and plan for contingencies that may affect CDS adoption and use.

Reports of CDS effectiveness in the literature in general have been mixed [8, 26]. A recent systematic review of by Van de Velde et al. found that CDS adherence was improved through computerized automation, making the CDS more patient-specific, and providing CDS to patients rather than only to providers [26]. However, adherence was slightly improved for on-screen CDS displays compared with paper versions [26]. While the integrated cancer prevention and cardiovascular risk management CDS intervention is automated, tailors the CDS to the patient, is available electronically to PCPs in the EHR, and is designed for both patients and PCPs, it relies heavily on printed handouts. The original cardiovascular CDS on which the integrated CDS is based is a paper-focused computerized CDS that has been shown to be effective in clinical practice [9]. However, the research team is working on developing CDS that minimizes the use of paper for use at the healthcare system post-intervention.

More generally, CDS does face adoption barriers [7]. To boost CDS intervention uptake, Medlock et al. proposed the “Two-Stream Model,” a framework combining both patient and provider CDS interactions [27]. CDS developers could use this model when assessing CDS effectiveness, including the ability of CDS to enhance shared decision-making between patients and PCPs. As our interviews with key informants showed, the presentation of CDS as described by Medlock [27], including content, timing, channel, format, notification, and interaction functions, may be key user areas that can make or break CDS success. They are also related to three of Bates et al.’s “Ten Commandments for Effective Clinical Decision Support” in medicine that developers of CDS should be aware of and address: speed, integration into clinic workflow, and forced stops [28]. The pre-implementation design of the cancer prevention and cardiovascular risk management CDS focused specifically on enhancing these areas.

The GUIDES checklist, published after we had completed our pre-implementation key informant interviews, may be a timely and useful checklist for implementing guideline-based CDS [29]. The GUIDES checklist was developed based on opinions from international CDS experts, patients, and other healthcare consumers. It encompasses four domains: context related to potential CDS success; CDS content; the CDS system; and implementation of the CDS into practice [29]. While a unified framework for developing CDS does not yet exist [7], having tools like the Two-Stream Model and the GUIDES checklist in hand when planning both the development and the implementation of guideline-based CDS may help researchers create CDS best suited for its intended purpose and audience.

Based on feedback from extensive pre-implementation site engagement, pilot testing, and key informant interviews, we made several changes to our study protocol and the intervention design to better adapt to the healthcare system’s needs and culture. These changes are being reported on separately. In brief, we offered multiple training modes and learning points and conducted in-person training sessions at all study intervention clinics. Training also included content on the CDS’s potential impact on quality measures and patient health, team model of care, recommendations based on the latest evidence [10–14], and alignment with the healthcare system’s aspirational aims. Concerning CDS design, the research team initially worked with a design firm to make changes to the CDS interface to incorporate all cancer prevention and cardiovascular CDS domains. After initial designs were completed, the research team took over making changes to the CDS interface during pilot testing and has continued to make changes based on PCP feedback as needed since the intervention went live in May 2018. The CDS is meant to be adaptable as recommendations change and new health priority areas are added.

We are also undertaking several steps to address potential barriers. The CDS is available to RNs conducting Medicare annual wellness visits. We recommend the CDS’s “Suggested Improvements” feedback button as the quickest way for PCPs and rooming staff to directly bring issues to the research team’s attention. Research team staff also communicate regularly with intervention clinic leaders and share monthly clinic and PCP-specific print rate reports. Moreover, we found that two key informants had doubts about the HPV vaccine and another distrusted risk calculators. These PCP reservations are important to note and address through intervention training, as they could affect CDS uptake. The HPV vaccination decision aids and cancer risk calculators built into the CDS may also educate some PCPs and patients.

Other dissemination and implementation science efforts planned include conducting post-implementation patient and key informant interviews. We conducted a pre-implementation PCP survey and will administer a follow-up PCP survey in later study years. We also recently conducted a post-implementation patient survey.

### Limitations

Results are limited to key informants who agreed to take part in interviews and were employed by the healthcare system. Informants other than those interviewed may have had different responses than those reported here. Furthermore, key informant age and years in practice at the time of pre-implementation interviews were unknown, limiting our ability to assess the potential for adoption based on these PCP characteristics. However, we will collect this information on CDS users going forward post-implementation, which will allow us to assess their influence on CDS adoption. Also, the design of the CDS printouts changed between key informant interviews and implementation of the CDS. However, the new design provides the same guideline-supported CDS content. As noted by Fraccaro et al. in a systematic review, few studies have assessed the impact of CDS systems with patients with multimorbidity; the conditions most frequently assessed together are cardiovascular risk and diabetes [30]. The current study focuses on integrating cancer prevention CDS, which targets all eligible patients, into a cardiovascular risk management CDS system that currently targets only patients with prediabetes, diabetes, or serious mental illness. CDS has an opportunity to enhance care for patients with multiple chronic conditions. More research is needed on CDS and multimorbidity and the interactions of care processes for this population [30]. Finally, this paper focused only on key informant opinions before implementation of the integrated CDS. Patient and key informant interviews are planned to assess opinions of and experiences with the cancer prevention CDS post-implementation.

## Conclusion

EHR-linked and web-based CDS for patients and PCPs may facilitate cancer prevention and screening shared decisions in primary care. Interviews with healthcare system key informants showed barriers and facilitators related to CFIR Intervention Characteristics, Inner Setting, Outer Setting, and Characteristics of Individuals domains. Our findings show the importance of assessing key informant input before CDS implementation. Findings also suggest that addressing PCP time limitations and EHR alert fatigue, among other barriers, may be necessary for successful CDS implementation and adoption. Maximizing workflow, incorporating team models of care, and offering multiple training modes, particularly in-person training, while emphasizing patient, provider, and organizational benefits may help facilitate cancer prevention CDS use in busy primary care practices. Post-implementation efforts may also be needed to ensure that identified barriers are successfully mitigated and to address any new CDS use barriers that arise.

## Additional file

Additional file 1: Stakeholder interview guides. Stakeholder interview questions used in this study. (DOCX 27 kb)

## Data Availability

The data analyzed during the current study are available from the corresponding author on reasonable request.

## Abbreviations

CDS: Clinical decision support
EHR: Electronic health record
HPV: Human papillomavirus
PCP: Primary care provider
RCT: Randomized control trial
RN: Registered nurse
USPSTF: United States Preventative Services Task Force
