# Supplementary material for: Barriers and facilitators to implementing cancer prevention clinical decision support in primary care: a qualitative study
Source: BMC Health Serv Res. 2019 Jul 31;19:534. doi: 10.1186/s12913-019-4326-4 (PMC6668099; doi:10.1186/s12913-019-4326-4)
Supplement: Supplementary file 1 — Stakeholder interview guides. Stakeholder interview questions used in this study. (DOCX 27 kb) [file 12913_2019_4326_MOESM1_ESM.docx]

Cancer Prevention Wizard Study

**Provider Key Stakeholder Interview Tool**

(*CFIR Domain: CFIR Construct*)

1. (*Characteristics of Individuals: Knowledge & Beliefs about the Intervention*) Prior to our presentation, where have you heard about the Cardiovascular Wizard?

PROBES:

- Daily Dose, primary clinic EMG meeting, clinic section meeting?
- What have you heard about it?
- What do you like about it?
- What concerns do you have about it?
- What could make it better?

1. *(Inner Setting: Tension for Change)* What are your initial thoughts on a single Wizard that assesses both a patient’s cardiovascular risk and cancer screening and prevention needs?

PROBES:

- - - Benefits or disadvantages to patients? To providers?
    - Integrating into clinic workflow?
    - Time considerations?

1. How would you prioritize cardiovascular risk and cancer screening for patients?
2. (*Intervention Characteristics: Relative Advantage*): In terms of cancer prevention, how does the Wizard compare to other Epic tools used at Essentia Health primary care clinics?

PROBES:

- What advantages does the Wizard have compared to these other interventions?
- What disadvantages does the Wizard have compared to these other interventions?

1. (*Characteristics of Individuals: Knowledge & Beliefs about the Intervention*) Do you think the Wizard will be effective in cancer screening and prevention in primary care at Essentia? Why or why not?
2. (If previously used the CV Wizard, else skip to Q. 8) How do you currently utilize the CV Wizard print-out during a patient visit?

PROBES:

- How do you get the print-out?
- Do you review the print-out before you go into the room?
- Do you review the print-out with the patient first?
- How do document your use of the tool in EPIC?

1. How will the Wizard affect Essentia primary care providers and staff in practice? How might it affect Essentia patients?

PROBES:

- Potential benefits?
  - Helping to meet organizational goals (population health, patient experience, cost)?
  - Engaging and educating the patient?
- Disadvantages?
- BPA only pops up on provider screen; a Wizard is especially designed for the patient

1. What triggers you to discuss cancer screening and prevention with your eligible patients?
2. How comfortable do you feel making a shared decision with patients about cancer screening?
3. What are your thoughts on the cancer prevention shared decision-making tools that will be available in the Wizard for breast, lung, and colorectal cancer and HPV vaccination?

PROBES:

- Prioritizing?

1. Do you think the Wizard and the shared decision-making tools would help you make a shared decision with your patients? Why or why not?
2. (*Inner Setting: Implementation Climate*) Given your understanding and experience at Essentia, what do you feel are major or minor concerns we need to address to ensure a seamless implementation and high utilization of the Wizard? Do you have suggestions about how to best address or overcome these concerns?

PROBES:

- Barrier/Concern Examples (if needed):
  - Organizational barriers
    - Skepticism regarding new innovations, lack of support
    - Organizational structure and climate
    - Alert fatigue/competing demands
    - Cost, time
  - Study implementation barriers
    - How the study is introduced and implemented
      - Inclusion in planning
      - Training
  - Patient issues
    - Experience with information, shared decision-making

1. Is there anything that we did not talk about that you would like to mention?

PROBES:

- Additional comments?
- Additional questions?
- Additional concerns?

1. Lastly, would you like to continue to be updated and/or involved with this project? What is the best way to do that?

Cancer Prevention Wizard Study

**Leadership Key Stakeholder Interview Tool**

(*CFIR Domain: CFIR Construct*)

1. (*Characteristics of Individuals: Knowledge & Beliefs about the Intervention*) Prior to our presentation, where have you heard about the Cardiovascular Wizard?

PROBES:

- Daily Dose, primary clinic EMG meeting, clinic section meeting?
- What have you heard about it?
- What do you like about it?
- What concerns do you have about it?
- What could make it better?

1. *(Inner Setting: Tension for Change)* What are your initial thoughts on a single Wizard that assesses both a patient’s cardiovascular risk and cancer prevention screening needs?

PROBES:

- - - Benefits or disadvantages to patients? To providers?
    - Integrating into clinic workflow?
    - Time considerations?

1. What are Essentia’s organizational priorities and goals regarding cancer prevention?

PROBES:

- Facilitate patient-centered care?
- Facilitate shared decision-making?
- Provide patient-tailored and evidence-based recommendations at point-of-care?
- What are initiatives around cancer screening?
- Are there any Essentia wide programs around cancer prevention?

1. (*Intervention Characteristics: Relative Advantage*): In terms of cancer prevention, how does the Wizard compare to other Epic tools used at Essentia Health primary care clinics?

PROBES:

- What advantages does the Wizard have compared to these other interventions?
- What disadvantages does the Wizard have compared to these other interventions?

1. (*Characteristics of Individuals: Knowledge & Beliefs about the Intervention*) Do you think the Wizard will be effective in cancer screening and prevention in primary care at Essentia? Why or why not?
2. How will the Wizard affect Essentia primary care providers and staff in practice? How might it affect Essentia patients?

PROBES:

- Potential benefits?
  - Helping to meet organizational goals (population health, patient experience, cost)?
  - Engaging and educating the patient?
- Disadvantages?
- BPA only pops up on provider screen; a Wizard is especially designed for the patient

1. (*Inner Setting: Implementation Climate*) Given your understanding and experience at Essentia, what do you feel are major or minor concerns we need to address to ensure a seamless implementation and high utilization of the Wizard with physicians? With nurses? With other clinic staff? With patients? With Essentia leadership, including informatics and quality improvement staff?

PROBES:

- Do you have suggestions about how to best address or overcome these concerns?
- Barrier/Concern Examples (if needed):
  - Organizational barriers
    - Skepticism regarding new innovations, lack of support
    - Organizational structure and climate
    - Alert fatigue/competing demands
    - Cost, time
  - Study implementation barriers
    - How the study is introduced and implemented
      - Inclusion in planning
      - Training
  - Patient issues
    - Experience with information, shared decision-making

1. (*Outer Setting: External Policies & Incentives*) Can you tell us about any external factors that may affect implementation or use that we should be aware of?
2. What are your thoughts on the cancer prevention shared decision-making tools that will be available in the Wizard for breast, lung, and colorectal cancer and HPV vaccination?

PROBES:

- Prioritizing?

1. What kind of feedback have you received from clinic leaders about the Wizard?

PROBES:

- Positive feedback?
- Negative feedback?

1. Is there anything that we did not talk about that you would like to mention? Who else (show list) in Essentia would you recommend we talk with about implementing the Wizard? Why?

PROBES:

- Additional comments?
- Additional questions?
- Additional concerns?

1. Lastly, would you like to continue to be updated and/or involved with this project? What is the best way to do that?
